# Working young adults’ engagement with public and workplace health promotion efforts in Singapore: A qualitative study

**DOI:** 10.1371/journal.pone.0309983

**Published:** 2024-10-22

**Authors:** Jodie Leu, Salome A. Rebello, Ginny M. Sargent, Matthew Kelly, Cathy Banwell

**Affiliations:** 1 National Centre for Epidemiology & Population Health, The Australian National University, Canberra, ACT, Australia; 2 Saw Swee Hock School of Public Health, National University of Singapore, Singapore, Singapore; University of Hyderabad, INDIA

## Abstract

Young adults entering the workforce are at increased risk of becoming overweight or obese. Yet, internationally, young adults are rarely targeted in health campaigns, and little is known on how to improve their interest and participation in health promotion efforts. Through 33 semi-structured interviews with young adults working in Singapore, we explored their engagement with existing public and workplace health promotion efforts that encouraged healthy eating and increased physical activity, and their subjective health in the context of their daily lives. Interviews were coded and thematically analysed, taking an inductive and deductive approach. Despite some interest in health messaging, participating working young adults rarely adopted health-promoting activities such as meeting daily recommended servings of fruits and vegetables and exercising regularly. Participants reported that the health promotion campaigns they were aware of, suggested actions that they couldn’t incorporate in their lives as they did not address the barriers that they are facing from socio-environmental contributors such as long working hours, personal and social commitments, and the food environment. Furthermore, some considered their health to be good enough and therefore had little reason to change existing practices. Affordable and accessible health screenings were an objective way for young adults to re-evaluate their perceptions of their own health which motivated some to favourably change their behaviours. Our findings suggest that future health promotion campaigns would benefit from more fully addressing some of the existing barriers that young working adults face, in tandem with policy changes to directly tackle the socio-environmental conditions for young workers.

## Introduction

In Singapore, a high-income, multi-ethnic Southeast Asian country, obesity prevalence is highest amongst young adults aged 30–39 years (12.4%), almost double that observed in 18–29 year olds (6.6%) [1]. In most other settings obesity prevalence peaks in age groups between 40–74 years old [2–4]. Despite these differences, rapid weight gain is observed in young adulthood globally [4, 5], suggesting specific lifestyles and habits in young adulthood result in weight gain.

Transitions to adulthood include major lifestyle changes such as leaving the family home, starting careers, and forming families, all of which may influence long-term dietary and physical activity practices [6]. These transitions also increase the risk of obesity and associated non-communicable diseases due to consumption of calorie-dense, nutrient-poor foods high in salt and sugar, and insufficient exercise [7–9]. Supported by a diverse, accessible food environment, eating out is widespread, with 77.3% of Singaporeans usually having either breakfast, lunch, or dinner outside the home [10]. Additionally, between 42.9% [1] to 54.0% [8] of the population do not participate in any leisure-time physical activity. Dietary and activity behaviours are one of the leading causes of premature death and disability in Singapore [11].

Singapore has a long history of health campaigns, policies, and programmes, including health messaging and providing recreational areas to promote healthy lifestyles. There is a recognized need to target behavioural changes, particularly to control and prevent diabetes [12]. The government has introduced health promotion initiatives such as the Healthier Dining Programme aimed at identifying healthier food options at food outlets [13]. To promote physical activity, the Walk2Ride program that began in 2013, extended sheltered walkways to connect key developments near transport hubs, and developed island-wide cycling paths and park connectors [14, 15]. Singaporean policies limit the number of vehicles on the road to prevent congestion and promote liveability [16] while a highly efficient public transportation system encourages incidental exercise. Additionally, a slew of health campaigns was introduced as part of the “War on Diabetes” that targeted modifiable risk factors in the general population such as smoking and alcohol consumption in addition to obesity, physical activity and diets [12, 17]. Government grants are provided to businesses to promote workplace health and well-being [18]. Government-endorsed health campaigns targeting healthy eating and physical activity among young adults at the time of data collection of this study (2016 to 2017) are summarised in S1 Table. These campaigns were often disseminated with other government-endorsed health-related information through varied media such as posters in public spaces and social media. Ongoing government health initiatives may be viewed at https://www.hpb.gov.sg/.

Despite these health promotion activities, only 41.2% of 18–29 year olds participated in regular leisure-time physical activity and approximately a third of older age groups [1]. Among adults aged 18–39, full-time workers were less likely to meet minimum physical activity requirements in comparison to full time students who had more available time [17]. Main reasons cited for inactivity in Singapore were work and family commitments, lethargy, and no interest in sports [8, 19]. Additionally, Singaporean young adults are not meeting recommended fruits and vegetables consumption and have high sodium intake [20, 21].

Due to the focus on economic development, Singaporean workers have one of the longest average working hours in the world at 44.6 hours per week [22]. Working more than 40 hours per week impacts workers’ physical and mental health, contributing to obesity and related chronic health conditions by diminishing their ability and time for self-care in Australia [23–25] and the USA [26]. In addition, young adults generally perceived themselves as healthy, contributing to a lack of reflexivity about their health [27]. Furthermore, the effects of long working hours accumulate over the life course [28].

In other countries, such as the UK, young adults are not specifically included in health promotion efforts and are challenging to target as they are not interested in existing health messaging that do not address barriers towards healthy lifestyles [29]. There is also limited research into the efficacy of health promotion efforts for this population [17]. Despite the proactive stance taken by the Singaporean government, existing health campaigns do not specifically target young adults (S1 Table). Thus, this study explores working young adults’ perceptions and engagement with health promotion campaigns and messages that encourage healthy eating and increased physical activity in Singapore, and their subjective health in the context of their daily lives. The study findings may guide and inform future health promotion efforts, particularly in other urbanised cities in Southeast Asian countries.

## Methods

### Study design

This study took a qualitative approach to gain in-depth insights into young adult workers’ perceptions and experiences of health promotion efforts. Qualitative data collection can be flexibly used to provide detailed, in-depth descriptive data that can help identify new aspects and patterns of social interactions in their context, such as environmental and social structures and nuances in everyday life which quantitative data does not capture [30, 31]. While in general, young adults may encompass teenagers to the mid-30s, ‘young adults’ in this study refers to those in their early 20s to mid-30s. The research was part of a year-long focussed ethnographic study [32] exploring interactions between everyday lives of Singaporean young adults, and their food and health practices and implications for health-promoting practices. Other papers have been published from the same data set [28, 33].

Participants engaged in in-depth, semi-structured interviews, provided socio-demographic information, and answered standardised questionnaires on their time use and physical activities. The lead author conducted participant observation and wrote fieldnotes of public dining occasions which were used to inform her understanding of the information gained during interviews (see details below).

### Recruitment

A sample of 15 men and 18 women between 23 to 36 years old took part in the study. All participants were Singaporean citizens or permanent residents who had lived in Singapore and worked full-time or at least 38 hours per week at the same job for at least one year. Participants were recruited between June to October 2016 through two approaches: specifically, via purposive sampling and snowball sampling. Purposive sampling is a strategy use to deliberately seek out and recruit participants of appropriate age with lived experience to provide insight into the studied phenomena [34]. JL contacted research participants in previous studies conducted by the National University of Singapore (NUS) who had consented to be contacted for further research, and friendship networks, to recruit participants who fulfilled the criteria described above at arms-length. After initial contact, potential participants were e-mailed study materials [28]. Prior to participating in the interview, all participants were fully informed regarding the study and their right to refuse to answer any questions and withdraw at any time. Written informed consent was obtained from all participants. There was no prior relationship with the study participants. The first cohort of participants were asked to suggest other potential participants, who were then contacted to recruit in a “snowball” approach [30]. Of the 33 participants, 17 were recruited through the NUS cohort and 16 through JL’s contacts’ acquaintances.

### Data collection

The lead author, a female postgraduate student, trained in qualitative methodologies, with Asian heritage and of similar age to participants, collected all data for this study. She had previously lived in Singapore and had developed interest in Singaporean culture. Based on work by Eckermann [35], Hubert [31, 36], Mack [30], Bisogni [37–39], Blake [40], and Jastran [41], a semi-structured interview guide was developed and deployed flexibly to gain insight into participants’ understandings and beliefs related to perceptions of health; self-rated health; self-reported height and weight; food and dietary practices; perceptions of the foodscape; work and work culture, inclusive of working hours; time usage; physical activity; and broader socio-cultural influences such as family, friends, social media elements and government initiatives. Interviews were conducted in English along with a short quantitative questionnaire to capture demographic information; a global physical activity questionnaire; and a three-day time-use diary to record time spent on daily activities like cooking, cleaning and working. Participant observation occurred throughout a year of fieldwork focusing on food-related spaces such as hawker centres (a Singaporean-style food court).

Face-to-face interviews of approximately one hour were conducted at public locations and audio recorded. Field notes were taken after each interview. Interviews were conducted until data saturation occurred when no additional novel data were found [42]. The concept and practice of data saturation were discussed among all authors. None of the recruited participants dropped out of the study.

### Data analysis

Interviews were transcribed and the lead author read and reread the transcripts for familiarisation. To minimise the burden on participants member-checking was not used. Transcripts were analysed using Atlas.ti 8 Windows to facilitate the coding process. An inductive and deductive thematic analysis was used [43]. An inductive approach identifies relevant and informative text segment from transcripts which are then coded which may identify new information of the studied phenomena. A deductive approach attaches codes to text that reflect existing literature on the subject matter and is in line with a focussed ethnographic approach [43]. A tentative coding book was developed with initial codes identified from existing literature and novel codes identified from the transcripts. The codebook was then updated and amended several times as more transcripts were coded and new codes were identified [44]. All authors discussed key themes and concepts forming the coding framework. The codes were grouped into higher level categories to identify themes and reviewed further [43]. Quantitative data from questionnaires were analysed using Microsoft Excel to summarise descriptive demographic, work, and physical activity information. Metabolic equivalents (METs) were calculated following the GPAQ analysis guide [45]. The Consolidated Criteria for Reporting Qualitative Studies (COREQ) checklist was followed for this study [46] (S2 Table).

### Ethical approval

Ethical approval was attained from the Australian National University Human Research Ethics Committee (Protocol: 2015/813) and the National University of Singapore Institutional Review Board (B-16-076). To protect their anonymity and maintain confidentiality, pseudonyms were assigned or provided by participants. Upon completing research activities, participants were offered a SGD20 supermarket voucher to thank them for their time.

## Results

Thirty-three participants aged 23 to 36 (15 men, 18 women) participated in the study. Table 1 shows the sociodemographic characteristics of participants. The ethnicities of the participants reflect Singapore’s ethnic composition that is predominantly Chinese (74.3%), followed by Malay (13.4%), Indian (9.0%), and other ethnicities (3.2%) [47]. Participants worked for different sized businesses, ranging from being self-employed, small businesses with a handful of people, small medium businesses (some of which were local offices of multinational companies), to large businesses such as government departments and multinational companies.

**Table 1:** Sociodemographic information about participants.

	Total (N = 33)	Men (N = 15)	Women (N = 18)
	N (%^b^)		
**Ethnicity**			
** Chinese**	23 (69.7)	11 (73.3)	12 (66.7)
** Malay**	6 (18.2)	1 (6.7)	5 (27.8)
** Indian**	3 (9.1)	3 (20.0)	0 (0.0)
** Other**	1 (3.0)	0 (0.0)	1 (5.6)
	Mean ± SD		
**Age (years)**	28.7 ± 0.7	29.1 ± 3.2	28.3 ± 4.6
**BMI (kg/m** ^**2**^ **)**	22.3 ± 3.8	24.6 ± 3.0	20.5 ± 3.6
	N (%^b^)		
**BMI Categories**			
** Underweight < 18.5**	4 (12.1)	0 (0.0)	4 (22.2)
** Normal weight 18.5–24.9**	22 (66.7)	10 (66.7)	12 (66.7)
** Overweight 25.0–29.9**	5 (15.2)	3 (20.0)	2 (11.1)
** Obese ≥ 30**	2 (6.1)	2 (13.3)	0 (0.0)
**Self-rated health**			
** Poor**	2 (6.1)	1 (6.7)	1 (5.6)
** Fair**	7 (21.2)	2 (13.3)	5 (27.8)
** Good**	16 (48.5)	6 (40.0)	10 (55.6)
** Very good**	6 (18.2)	4 (26.7)	2 (11.1)
** Excellent**	2 (6.1)	2 (13.3)	0 (0.0)
**Level of physical activity according to GPAQ**			
** High**	6 (18.2)	3 (20.0)	3 (16.7)
** Moderate**	20 (60.6)	9 (60.0)	11 (61.1)
** Low**	7 (21.2)	3 (20.0)	4 (22.2)
** Meeting the equivalent of 600 MET minutes per week** ^**a**^	27 (81.8)	12 (80.0)	15 (83.3)
**Living arrangements**			
** With family**	28 (84.8)	12 (80.0)	16 (88.9)
** Housemates**	3 (9.1)	2 (13.3)	1 (5.6)
** Alone**	2 (6.1)	1 (6.7)	1 (5.6)
**Marital status**			
** Never Married**	23 (69.7)	10 (66.7)	13 (72.2)
** Married**	10 (30.3)	5 (33.3)	5 (27.8)
**Number of children**			
** 0**	27 (81.8)	13 (86.7)	14 (77.8)
** 1**	1 (3.0)	1 (6.7)	0 (0.0)
** 2**	4 (12.1)	1 (6.7)	3 (16.7)
** 3**	1 (3.0)	0 (0.0)	1 (5.6)
**Highest completed level of education**			
** Secondary**	1 (3.0)	0 (0.0)	1 (5.6)
** Diploma**	8 (24.2)	3 (20.0)	5 (27.8)
** Bachelor’s**	20 (60.6)	9 (60.0)	11 (61.1)
** Professional Certificates**	1 (3.0)	1 (6.7)	0 (0.0)
** Master’s**	3 (9.1)	2 (13.3)	1 (5.6)
**Occupation**			
** Legislators, Senior Officials and Managers**	2 (6.1)	0 (0.0)	2 (11.1)
** Professionals**	24 (72.7)	13 (86.7)	11 (61.1)
** Associate professionals and technicians**	1 (3.0)	1 (6.7)	0 (0.0)
** Clerical Support Workers**	5 (15.2)	1 (6.7)	4 (22.2)
** Service and Sales Workers**	1 (3.0)	0 (0.0)	1 (5.6)
**Actual hours worked per week**			
** <44**	11 (33.3)	3 (20.0)	8 (44.4)
** 44**	2 (6.1)	1 (6.7)	1 (5.6)
** >44**	20 (60.6)	11 (73.3)	9 (50.0)
**Monthly income inclusive of CPF (SGD)**			
** 1000–2000**	2 (6.1)	1 (6.7)	1 (5.6)
** 2000–3000**	7 (21.2)	2 (13.3)	5 (27.8)
** 3000–4000**	6 (18.2)	2 (13.3)	4 (22.2)
** 4000–5000**	12 (36.4)	5 (33.3)	7 (38.9)
** 6000–10,000**	6 (18.2)	5(33.3)	1 (5.6)

^a^ Participants who have met recommended 150 minutes of moderate intensity aerobic exercise or at least 75 minutes of vigorous-intensity aerobic exercise in a week [48] which is the equivalent of 600 MET minutes per week.

^b^Percentages may not add up to 100 due to rounding.

### Subjective and self-rated health

Health-related behaviour change is usually driven by a perceived need to initiate new healthy habits or quit existing unhealthy ones. The health belief model suggests that triggers resulting in the awareness of susceptibility or severity of a condition can lead to behavioural changes [49, 50]. Consequently, to consider young people’s acceptance and adoption of health promotion campaigns it is helpful to understand their perceptions of their health. When participants were asked to rate their health out of the following: poor, fair, good, very good, and excellent, nine (27.27%) participants rated their health as poor or fair; most, 16 (48.48%), rated their health as good; and eight (24.24%) participants rated their health as very good or excellent. Two men, but no women, rated their health as excellent.

Considerations that formed the basis of how participants rated their health, revolved around their weight, existing health conditions, and the amount of activities perceived as health-promoting. For instance, Laila (female, 23, nanny, living with partner and housemate, never married) rated herself as having poor health due to a lack of time from work and studies to monitor her weight which had increased. Even participants who rated their health as fair generally thought their health could be better if they actively incorporated one or more health promoting activities such as exercising more and eating healthy foods. For instance, Xin Yi (female, 29, editorial staff, living with parents, never married) thought she should eat more fruits and vegetables. Men who rated their health highly usually mentioned a combination of exercise frequency, healthy eating, and a lack of existing medical conditions. Meanwhile, overweight participants acknowledged that they should be aware of their health habits.

Generally, participants’ BMIs did not align with their self-rated health. A typical example was when a male participant considered himself to have good health although his BMI indicated that he is overweight. He attributed this to muscle mass. Meanwhile, some women rated their health as good or very good despite being classified as underweight. Many women described better health in the context of lower weight and a few women countered weight gain by limiting their food intake, including having only two meals a day. Participants who had a family member with health conditions were somewhat more health conscious and proactive. Older participants were more concerned about their health. Some participants reported phases of eating healthy and exercising due to embodied feelings or their appearance. All participants, regardless of their self-rated health, felt they could improve or incorporate more health-promoting habits to be healthier.

### Engagement with health campaigns

Government-sponsored health campaigns (S1 Table) contributed somewhat to participants’ ideas of health and health promoting practices. Participants frequently named the Healthier Choice Symbol and the ‘Ask for’ programme when asked about their experience with health campaigns. The ‘Ask for’ Programme encourages consumers to ask for more vegetables and less oil, salt, sugar and syrups when eating outside of the home (Health Promotion Board 2017). Participants commonly repeated government-endorsed health information during interviews. However, participants said these had a limited impact on them. For example, most were aware of the Healthier Choice Symbol, but it did not strongly influence what food products they purchased. Laila exemplified a commonly held view (female, 23, nanny, living with partner and housemate, never married) saying, *“it’s there*, *but it’s useless*, *you know*? *It’s like “yeah yeah I know”*, *you know you can ask anyone… but they will never do it”*. Alex (male, 28, internal auditing, living with parents, never married) explained it this way “*I’m aware of [healthier] choice symbols…it doesn’t affect how I decide… I just eat what I want to*, *so I just don’t really look up for Healthier Choice Symbols*”.

Other participants perceived their diets to be healthy enough particularly when incorporating vegetables into food habits. Hence, Healthier Choice Symbols and related healthy diet campaigns made little difference to them because they felt confident in their knowledge about healthy food.

*…my diet is actually is pretty, pretty much healthy because I don’t take a lot of oily stuff? Or those with a lot of fats… I just don’t like it… and I’m more of the soupy kind of person so I don’t really take a lot of fried food, barbecue food… So I can quite safely say that I can ignore all these Healthier Choice [Symbols]? Cause anyways, I take a lot of grains… and I’m confident to say I eat a lot of fruits… . –Andrea, female, 24, human resources, living with parents and sibling, never married*

The purchase of healthier items for cooking and consumption at home was irrelevant to the substantial proportion of participants who lived in the family home and were not involved with purchasing groceries and food preparation. Conversely, participants with children who cooked at home were more likely to choose healthier items such as cooking oils displaying the Healthier Choice Symbol.

Workplace-based health promotion is a key element in the government’s strategy. The government provided grants for health promotion activities conducted by businesses [18]. Larger and wealthier private and public sector workplaces offered a variety of health promotion activities such as annual health checks and organized activities including optional workshops, seminars, and exercise classes while some participants worked in multinational companies that provided comprehensive workplace health promotion as part of the work package.

One of the well-received health-promoting activities in larger private and public sector workplaces was the annual health check-up which usually included measurements of BMI, waist circumference, blood pressure, fasting venous blood glucose and lipid profile. Additional check-up items were sometimes provided by companies or self-paid by participants or their private health insurance. If participants worked in medium enterprises, they self-paid for health checks, which was a major disincentive. Self-employed participants and participants who worked in small companies did not recall any health-related initiatives in their workplaces.

People generally found the annual health checks helpful. For example, Dhruv (male, 29, public service, living with flatmates, never married), changed his diet when a complimentary annual workplace health check showed he had elevated cholesterol levels. For Uzma, she transitioned to a healthier lifestyle after seeing her weight increase and her doctor’s comments.

*[I] was close to [60 kgs], so that was like a wakeup call, so that’s when I decided to exercise… [and] I think it was cholesterol, was on borderline high, and the doctor mentioned that I need to change my diet, yeah so that was also when I decided to, need to change… I was like most young adults when they first start out in their twenties, they think like " my twenties can last another 10 years"- Uzma, female, 34, property executive, living with a parent, never married, also has family health conditions that contributed to being health-conscious*

Uptake of workplace annual health checks was dependent on whether participants were allocated time during working hours (as opposed to needing to take personal leave) and the proximity of the healthcare service. Depending on how comprehensive the health checks were, some could take up to half a day. Some participants chose to forgo health checks because they were fearful about what might be found or were disinterested. In addition to health checks, some workplaces offered health-related talks and workshops, but attendance was based on participants’ time and interest.

These talks could impact health-promoting activities amongst participants. For example, one woman recounted how a hands-on workshop on healthy cooking and reading nutrition labels at the supermarket helped her make healthier choices for herself and her whole family. After the workshop, she switched to lower saturated cooking oils for home cooking and purchased healthier items, aided by the Healthier Choice Symbol health campaign.

Often, workplaces provided fruit baskets. However, this did not seem to increase participants’ consumption of fruits as many did not like the offered fruit or could not be bothered to eat it. Regarding physical activity, some participants mentioned corporate sports days or organised group exercise events. Group activities tended to focus building and maintaining social bonds in the workplace. Again, participation was dependent on existing bonds within the workplace. Lim (male, 33, human resources and statistics analyst, living with a parent, married) recalls successfully encouraging a group of co-workers to go on weekly runs with him which stopped when he was relocated. Meanwhile, participants tend to avoid group activities attended or organised by bosses as they did not want more opportunities to talk about work or did not enjoy their bosses’ company. At Andrea’s workplace, the bosses led by example in taking a compulsory stretch or walk break at a designated time every day for about five or ten minutes.

*Because it wasn’t top-down initially, it was by my team… but no one seemed to do that because they can … so now the boss says “[designated time] o’clock”, everyone is like “ok [designated time] o’clock”. So, our bosses walk and talk, they walk the talk.–Andrea, female, 24, human resources executive, living with parents and sibling, never married*

Most participants had some awareness of the physical activity related health promotion campaigns, although they too had little long-term influence. For instance, Kenneth (male, 27, banking, living with a grandparent, parents, sibling, and helper, never married) remarked that there has been an increase in marathons organised by the government and commercial entities, while Rashid (male, 26, accountant, living with parents, never married) recalled seeing shopping mall workouts, however, they rarely engaged with these events. The ActiveSG promotion (S1 Table) received similar reactions. For instance, Nadia (female, 23, pharmacy technician, living with parents, never married) and her colleagues exercised at the facilities offered by ActiveSG after work, but stopped after a few months with Nadia remarking *“So all our energy has been drained [at work]*, *we just want to go home and rest”*. Other participants found the location of ActiveSG facilities inconvenient. Some participants and their families and colleagues engaged with the National Steps Challenge of their own volition or were encouraged by their workplaces, although how long they engaged with the challenge depended on whether they lost the steps tracker and their level of interest.

Group physical events after work or on the weekends were less popular due to workers’ social and family commitments. Participants who were married, especially those with children, rarely took part in after-work activities due to household tasks and childcare. Some employees had access to a gym at work, which may even be within a five-minute walk. While some participants utilised the gym during lunchtime, most did not as they may prefer socializing with co-workers over lunch, felt it was inconvenient to shower and change during the workday or felt lunchtimes were limited in duration to accommodate exercise.

### Complexities in adhering to healthy eating messages

Few unusually health-conscious participants met the daily dietary requirements. Other participants consistently expressed they were aware of messages about healthy eating but generally did not follow them. Instead, many followed the examples set by family and friends, social media, and the internet. They generally consumed at least some fruits or tried to consume less seasoning, salt, and oil. Their reasons for not complying with health messages ranged from lack of self-control, disinterest, dislike of “healthy” foods and lack of time. They found it easier to meet their daily requirements of vegetables than fruits or to reduce their intake of unhealthy food.

*What could I realistically do… I think if I can’t supplement myself with very healthy and good food then, what I can do is cut down on unhealthy food. I think that kind of balances it off right? Like instead of always snacking on chips and all like that, I could eat something else better, I guess?- Adeline, female, 28, account management, married but living at home with parents*

Many participants often ate meals at home with their family, so had little control over food purchasing and preparation which was usually done by parents or grandparents. Although home-cooking is generally considered healthier than commercially prepared food [51], some participants blamed home-cooked meals for their weight. The reasons ranged from household preferences for heavily seasoned foods and eating more than needed because the food was tasty and cultural practices where family members show care through providing food.

*There’s a lot of vegetables in Malay cuisine but it’s always cooked in chili or curry, it’s never like cooked by itself. Or otherwise it’s like deep fried, like… not even in batter but it can just be fried…but the thing is that if [mum]’s the one cooking, she usually doesn’t listen, she’s just like "but you know, your dad likes it" so … "Don’t complain, you’re not the one cooking"–Sana, female, 23, financial public relations, living with parents and siblings, never married*

Conversely, when participants took an active role in meal preparation, they were more likely to increase their fruit and vegetable intake.

*I make sure that every day, we eat fruits, and also the vegetables, if I don’t get it during lunch, I will, I make sure that I get it, a big serving, during dinner. Because we cook ourselves, so we can cook whatever we like. –Hui Ting, female, 36, chemist, living with spouse and three children under five, married*

It was common for participants to eat at least one daily meal outside the home, where they have minimal control over food portion sizes. Despite government health campaigns to improve the healthfulness of food in hawker centres, participants frequently ate meals with little or no vegetables. At hawker centres that participants frequented, meal expenditures were usually below SGD5, which they considered affordable, however, extra serves of vegetables increased the cost of food and depended on whether the patronised food stall served vegetables.

Hawker stalls tend to sell a small menu of rapidly produced, inexpensive food. Participants expressed reluctance to ask for meals with extra vegetables or less oil and seasoning to avoid disrupting the hawkers’ food preparation and serving practices, especially during rush hours.

Compared to hawker centres, food outlets that served healthier foods tend to be considered beyond the daily budget for meals. Uzma recalled her experience regarding the cost of healthy food options where minimally processed foods are available:

*When you go outside to buy, they have those special shops that sell all healthy food. Yea but when I look at the price, it’s actually very expensive, like one plate or one box of what they call clean lean meat or whatever, it can cost like SGD19 –Uzma, female, 34, property executive, living with a parent, never married*

Cultural meanings attached to the meaning of healthy foods sometimes evoked thoughts of ‘sick people food’, meaning it was expected to be a tasteless, restricted diet or as one person said: *“Healthier food always tastes like crap*.*”*. These perceptions applied to the healthier options recommended by Health Promotion Board campaigns.

*Ok there are hawker stores that have these stickers on their glass boards. Ask for more vegetables, ask for less sugar, less cholesterol… Why would I want to? … If I ask for less sugar, less salt, my food will taste not so good? If I ask for more vegetable, I will be charged for it.- Lim, male, 33, human resources and statistics analyst, living with a parent, married*

### Difficulties in adopting physical activity guidelines

Overall, participants had minimal engagement with health promotion campaigns promoting physical activity. Despite government efforts to provide well-paved streets and sheltered walkways, Singapore’s hot, humid climate deterred participants from outdoor activities, especially when in work clothes. People were concerned about their appearance at work where tidiness is valued. Despite government efforts to encourage cycling by promoting bicycle lanes and facilitate cycling to work by adding showers in workplaces, only a few participants cycled during their leisure time and to travel to locations near their homes.

Many participants had adequate incidental activity, although most spent long periods of time being inactive. During the weekdays, they spent most of their time either sitting at a desk at work (daily average 8.7–9.0 hours), mostly sitting or standing during commutes (1.9–2.4 hours), or relaxing alone during leisure time such as looking at online content (0.9–1.1 hours) [28]. Work-related fatigue and exhaustion led participants to unwind and de-stress by socialising with family and friends, often over meals, or going on the internet. Participants with young children had less time for physical activity. However, a few participants exercised to gain and maintain a certain level of fitness or to reduce stress. They ran, exercised at home, or joined private gyms and classes. Some made use of workplace exercise events and facilities and public facilities to engage in physical activity with their friends. Some men proactively took part in physical activity, particularly in the preceding months, to pass their annual post-conscription fitness tests.

## Discussion

As insufficient physical activity and poor dietary intake are among the leading causes of premature death and disability in Singapore [11], there is pressure to improve population health outcomes. Overall, this study found that most of participants rated themselves as having good health. Consequently, they did not consider government and workplace health promotion campaigns around healthy diets and physical activity to be largely relevant to them. Furthermore, they found it difficult to improve their diets and physical activities due to social and environmental conditions. They argued that their long work hours among other elements encouraged the purchase of commercially prepared foods, while their physical activities were also limited by work demands and Singapore’s tropical climate. Affordable and accessible workplace health screening could provide objective assessments to help participants re-evaluate their own health and at times spur health promoting behavioural changes.

Participants’ accounts reveal the complex interplay of considerations contextualised within young Singaporeans’ everyday lives and how these affect their limited engagement with health campaigns and adoption of government health promotion advice. Other literature similarly finds that, in general, when young adults rate their subjective health positively and are unconcerned about the healthiness of their lives, health promotion campaigns have limited efficacy as they do not consider health promotion materials to be relevant to them [27, 52–54] even though the government is thought to be authoritative and truthful source of information [55].

A study of Singaporean university students found that 27.4% of young adults had prehypertension of which they were unlikely aware [56], and each additional meal eaten away from home per week raises the odds of prehypertension [56]. Additionally, global studies have found that markers of ill health in young adulthood may be silent, such as with hypertension and type 2 diabetes [56–58]. This is important as Asian populations are genetically predisposed to diabetes [58]. Hence, the evidence suggests that there are two dynamics at play. One is young adults’ unawareness of future health risks associated with their diets and physical activity. Judging their health to be reasonably good, they are unconcerned by future health problems. The second are the barriers to modifying their daily routines to incorporate healthier eating and more physical activity in the context of their daily commitments.

Regardless, our participants’ were aware of health promotion messages and thought that they should incorporate more health-promoting practices in their lives. Bombak [53] found that poor self-rated health status did not necessarily mean that people changed behaviour to improve health. Similarly, the habits of young adults in the US do not incorporate health-promoting practices [54]. How much importance young adults place on health and resultant health-promoting practices appears to be mainly influenced by their perception of the risk of ill health to them [27, 52]. For our participants, personal and indirect experiences with health conditions, a sense of fatigue and discomfort with their body and weight influenced their perceptions of ill health and future health risks which motivated them to take proactive measures. For most participants, despite having an awareness of government health information there was low efficacy in adopting these practices. Similar to Scottish young adults [29] our participants did not find the promoted information useful as it did not provide practical information on how to overcome barriers and difficulties that impede young adults from actively adopting healthy eating and physical activity practices.

Workplaces are an established setting for health promotion. However, similar to Australian workplaces, there was a lack of health promotion activities in small and medium enterprises and workplaces [59]. Between 2006 to 2010, health promotion programs in Singaporean workplaces with 5 to 49 staff increased from 0% to 27%, while over 50% of workplaces with 50 or more staff had health promotion programs [60]. In larger enterprises, there are more health promotion efforts by the management [61]. When offered, workplace annual health check-ups are popular among young adults to help them monitor their health. Health check-ups are important as early diagnoses can spur changes to diet and physical activity and improve prognosis [27, 52]. Although the Singapore Health Promotion Board offers a variety of health promotion activities and programs for different workplaces, subsidised health screening may not be available at all workplaces and for all young adults as eligibility is limited to citizens and permanent residents who are at risk of chronic conditions as identified by the Diabetes Risk Assessment tool and have not been screened in the last three years [62]. More recently, the Singaporean government has rolled out a national screening program to encourage regular health screening among eligible citizens for cardiovascular disease, cervical cancer and colorectal cancer at selected clinics at SGD5 or less [63]. However, in our study, uptake depended on whether young adults are allocated time within working hours and the location of participating health services. Additionally, other activities such as group-based sports outside of working hours had low engagement as young adults may be disinterested, instead prioritising social and family commitments and recuperation from work. A study of after-work health programs in Denmark showed low social support, especially when jobs had high physical and emotional demands that were out of workers’ control [64]. Australian workers were also reluctant to spend non-paid hours at workplaces when it cuts into their personal time and commitments [59, 65].

Most Singaporeans eat at least one meal away from home per day [10] and each additional meal eaten away from the home increases the likelihood of developing prehypertension [56]. Hence, there is a continued need to improve the nutritional quality of food sold at food vendors. For instance, sustained support for the use and development of healthier ingredients in the food service industry to promote normative use [12, 13, 66]. An intervention evaluating the Healthier Dining Programme at a tertiary institute found consumers more likely to have at least one healthier option meal when eating out due to food service providers cooking with healthier oil blends and lower-sodium salt [67]. A recent Singaporean study found that young adults exposed to health campaigns were more likely to meet dietary recommendations for fruit, vegetable, and wholegrain intake. However, given the cross-sectional study design, it is not clear if exposure to the campaign led to behaviour change or if people who had healthier habits were more likely to notice health related campaigns [17]. Concurrently, negative perceptions around the taste of healthy foods should also be addressed to improve acceptance of healthier ingredients and cooking methods. As the Healthier Choice Symbol has seen some success amongst our participants and tertiary students who are looking to eat healthier [68], continued emphasis could help promote healthier meals and ingredients for home use where young adults may have less control over prepared foods. Mandatory front-of-pack labelling of unhealthy beverages based on their sugar and saturated fat content, and advertising bans on the least healthy beverages (Nutri-Grade labels) are more recent environmental interventions by the Singapore government to help sugary beverage consumers make informed choices [69–71]. Extending similar interpretive labelling to foods may help mitigate potential health-halo effects of endorsement style labels such as the Healthier Choice Symbol [72].

It is imperative that more physical activity is promoted [73, 74], especially as most participants have relatively sedentary ways of living [17, 75]. However, existing initiatives have low uptake as they do not address barriers such as low interest in physical activity, family commitments and long working hours, especially as most participants (60.6%) worked more than 44 hours per week. Working women had less time than men due to gender disparities in childcare and domestic chores [76], a finding that is also common amongst mothers in our study [28]. Our findings corroborated with a local sports participation survey which showed that amongst young adults aged 20 to 39 who did not participate in physical activity, 61% reported a lack of time due to work, 47% were not interested in sports, and 29% reported a lack of time due to caring for family [19]. Despite expanding existing park connector networks and green space [77], participants are deterred by Singapore’s tropical climate and outdoor physical travel competes with the public transport system, which is much faster and more convenient, especially when participants must look work appropriate during working hours [15].

Overall, we found that both public and workplace health campaigns would benefit from considering the patterned, routine, and habitual ways that people live their lives [78], which leave participants with little time and energy for adding health-promoting practices especially when long working hours lead to time scarcity for self-care and health maintenance [23, 24, 79]. Health campaigns that focus on individual choices instead of generating broader societal changes, so that the ‘burden’ falls on individuals to be responsible for their health and engage in health-promoting practices are limited in effectiveness [80–82]. There is increasing evidence that a systemic and multilevel approach is necessary to target the promotion of these habits from multiple angles for population-level change directed towards the socio-environmental determinants of behaviour [12, 66, 83–85]. There are promising environmental interventions in Singapore underpinned by political support, platforms for government and food sector interactions, receptivity to national needs, monitoring of non-communicable disease risk factors, and dedicated funding for health promotion [70]. More recent interventions by the Singapore government such as the trans-fat ban and mandatory labelling of unhealthy beverages represent important steps to improve the food environment, but key concerns related to food labelling and nutritional quality of hawker foods persist [69–71]. Our results suggest that more robust implementation of the Healthier Dining Programme and effective labelling for food and out-of-home meals to improve food choice around commercial prepared foods [67, 70] may be supportive of food choice for young adults.

With flexible work arrangements a neccesity due to the Covid-19 pandemic, the Singaporean government has encouraged workplaces to sustain and promote these arrangements as a permanent feature in workplaces [86]. Before the pandemic, around 53% of Singaporean workplaces offered at least one formal flexible work arrangement, an increase from 47% in 2014 [87]. Previously, among private sector employees in Singapore, 2.9% were on office-based flexible work arrangments and 0.1% were on flexiplace arrangements in 2000 [88]. While there has been a momentum building in Singapore towards the incorporation of flexible work arrangements, the potential detrimental effects of such working arrangements on health need to be addressed.

Australian studies showed that even with flexible work arrangements, work demands and pressures that led to long working hours disrupted employees’ sense of control over their jobs and daily life and had negative impacts on well-being and health [89, 90]. Although flexible work arrangements are thought to help workers balance work with family commitments and engage in more health-promoting behaviours, any positive health effects depend on whether the arrangements meet workers’ needs, allow workers to have control over their time management, establish temporal routines that address blurring between work-time and home life, address stigma related to flexible working arrangements and working hours [91]. Flexible work arrangements driven by employee needs are popular and help facilitate work-life balance and family commitments, especially for women [89, 92], although flexible work can also interfere with family life [92, 93]. Amongst Singaporean mothers, the expectation to work beyond formal working hours to meet their contracted hours of work interferred with family life [93]. For white-collar Canadian working young adults, work demands and expectations took precedence on their time-use, such as working additional hours to meet deadlines; refraining from working from home to avoid negative impressions; and working longer hours at home due to expectations of availability that is not expected of those who worked in the office [94]. Flexible work arrangements also do not address barriers towards physical activity such as a lack of time, feeling tired, and not having motivation and employees were found to report less physical activity on days with flexible working hours [94, 95]. There remains a need to reduce and regulate working hours to help workers have more time for self-care, inclusive of healthier diets and more physical activity, and personal commitments as time is a finite resource. These aspects should be considered as Singapore’s national guidelines on flexible work arrangements come into effect in December 2024 as the nation transitions towards work from home arrangements [96].

### Limitations

The findings of this study do not reflect the conditions of all working young adults in Singapore or in non-metropolitan areas. Due to small sample sizes, qualitative research results cannot be generalised to broader populations. Instead, this study provides an understanding of why people of this age, working in these conditions, struggle to embed and maintain health promoting practices in their daily lives. These findings may well be relevant to other similar populations. Our participants were primarily white-collar workers and there were fewer participants who were obese than in the general population [1]. This could be due to self-selection bias as the study may have appealed more to young adults who are health-conscious. Nevertheless, the study recruited participants from a breadth of jobs, industries and workplaces to provide insights into a range of workers’ experiences of workplace health promotion. Future research could further investigate social and cultural influences impacting on population nutrition and physical activity over the life course.

Overall, this study provides insight into, and complements existing literature on, young adults’ engagement with public and workplace health promotion efforts and their perceptions of health in an urban Asian context.

## Conclusion

We found that while young workers are aware of health promotion campaigns, there are a range of personal, social and environmental barriers to their active engagement with health promoting practices. Health promotion campaigns could include affordable and accessible health screening but also need to acknowledge the specific influences that impact their daily lives and include intervention elements that are feasible and practical for them in light of their long and intense working hours.

## Supporting information

S1 Table Examples of population-wide Singaporean health campaigns targeting diets and physical activity inclusive of young adults between 2016–2017. (DOCX)

S2 Table Consolidated Criteria for Reporting Qualitative Studies (COREQ) checklist. (PDF)
