# Supplementary material for: Working young adults’ engagement with public and workplace health promotion efforts in Singapore: A qualitative study
Source: PLoS One. 2024 Oct 22;19(10):e0309983. doi: 10.1371/journal.pone.0309983 (PMC11495610; doi:10.1371/journal.pone.0309983)
Supplement: S1 Table — (DOCX) [file pone.0309983.s001.docx]

S1 Table. Examples of population-wide Singaporean health campaigns targeting diets and physical activity inclusive of young adults between 2016-2017.

| Health Campaign | Purpose | Activity |
| --- | --- | --- |

| National Steps Challenge [1, 2] | To encourage Singaporean residents (citizens and permanent residents aged 17 and above) to be more physically active. | Participants are gifted a pedometer if they don’t have one, and for each day that they accumulate steps, with a goal of 10,000 a day, prizes can be won. |
| --- | --- | --- |
| Healthier Choice Symbol [3] | The introduction of Healthier Choice Symbols on packaged food products and cooked foods to empower individuals to make informed healthier choices in their food practices. Food products follow guidelines on what can be labelled as a healthier choice. | A series of symbols to identify healthy options at supermarkets and food outlets. |
| ActiveSG [4] | To encourage physical activity by using sporting facilities at ActiveSG sports facilities located throughout Singapore. | Singaporeans are credited with SGD100 each year in their accounts that can be used towards booking facilities for group sports or gym passes for a workout session at SGD2.50 each time (Singapore Sports Council 2017, Singapore Sports Council 2017). |
| Health Promoting Malls [5] | A partnership with mall operators to promote products, services, and activities to improve the health and well-being of the local community which includes:   - Regular mall workout sessions led by fitness instructors - Healthier dining options at food outlets - Healthier Choice Symbol products endorsed at supermarkets - Professional advice at pharmacies for smoking cessation | A series of free exercise classes are offered at various participating shopping malls on a first-come, first-serve basis. |
| Healthier Dining Programme [6] | Encourages food and beverage businesses to provide healthier food and drink options. Eligibility to participate is dependent on meeting guidelines based on food outlet type and food products offered. | Provide consumers with healthier food and drink options, such as dishes made with healthier ingredients. Items are also labelled with the Healthier Choice Symbols to aid visibility. |

Note: Some health campaigns identified in this study crossed over with health campaigns within the broader War on Diabetes campaign that was launched in 2016 targeting modifiable risk factors associated with diabetes [7].

References

1. Ministry of Health Singapore. *National Steps Challenge Season 4 is here!* 2019 [cited 2019 6 June ]; Available from: <https://www.healthhub.sg/programmes/37/nsc>.

2. Chew, L., et al., *Can a multi-level intervention approach, combining behavioural disciplines, novel technology and incentives increase physical activity at population-level?* BMC Public Health, 2021. **21**(1): p. 120.

3. Health Promotion Board. *Healthier Choice Symbol*. 2019 [cited 2019 6 June]; Available from: <https://www.hpb.gov.sg/food-beverage/healthier-choice-symbol>.

4. Singapore Sports Council. *Membership*. 2017 [cited 2017 Dec 12]; Available from: <https://www.myactivesg.com/about-activesg/membership>.

5. Health Promotion Board. *Health promoting malls*. 2017 1 Mar 2017 [cited 2018 Jan 25]; Available from: <https://www.hpb.gov.sg/community/health-promoting-malls>.

6. Health Promotion Board. *Healthier Dining Programme*. 2021 16 May 2019 [cited 2022 23 June]; Available from: <https://www.hpb.gov.sg/healthy-living/food-beverage/healthier-dining-programme>.

7. Khow, Y.Z., et al., *Behavioral impact of national health campaigns on healthy lifestyle practices among young adults in Singapore: a cross-sectional study.* BMC Public Health, 2021. **21**(1): p. 1601.
